# Cyclic RGD-Functionalized and Disulfide-Crosslinked Iodine-Rich Polymersomes as a Robust and Smart Theranostic Agent for Targeted CT Imaging and Chemotherapy of Tumor

**DOI:** 10.7150/thno.37184

**Published:** 2019-10-17

**Authors:** Yan Zou, Yaohua Wei, Yinping Sun, Jie Bao, Feirong Yao, Zekun Li, Fenghua Meng, Chunhong Hu, Gert Storm, Zhiyuan Zhong

**Affiliations:** 1 Biomedical Polymers Laboratory, and Jiangsu Key Laboratory of Advanced Functional Polymer Design and Application, College of Chemistry, Chemical Engineering and Materials Science, and State Key Laboratory of Radiation Medicine and Protection, Soochow University, Suzhou, 215123, P. R. China; 2 International Joint Centre for Biomedical Innovation, School of Life Sciences, Henan University, Jin Ming Avenue, Kaifeng, Henan, 475004, P. R. China; 3 Department of Targeted Therapeutics, MIRA Institute for Biological Technology and Technical Medicine, University of Twente, PO Box 217, 7500AE, Enschede, The Netherlands; 4 Imaging Center, the First Affiliated Hospital of Soochow University, Suzhou, Jiangsu, 215006, P.R. China

**Keywords:** Targeted delivery, polymersomes, reduction-sensitive, solid tumor, CT imaging

## Abstract

There is tremendous interest in integrating CT imaging with chemotherapy; however, reported iodine-based nanosystems such as nanogels and nano-emulsions display typically reduced contrast coefficient, low drug loading and stability, and poor targetability. Here, cRGD-functionalized disulfide-crosslinked iodine-rich polymersomes (cRGD-XIPs) were designed as a novel, robust and smart theranostic agent and investigated for targeted CT imaging and chemotherapy of malignant tumors.

**Methods:** cRGD-XIPs were prepared from co-self-assembly of poly(ethylene glycol)-*b*-poly(dithiolane trimethylene carbonate-*co*-iodinated trimethylene carbonate) (PEG-P(DTC-IC)) and cRGD-PEG-P(DTC-IC) block copolymers. *In vitro* and *in vivo* CT contrast effect of cRGD-XIPs was studied using α_v_β_3_-overexpressing B16 melanoma as a tumor model in comparison with clinical agent iohexol. The therapeutic efficacy of doxorubicin-loaded cRGD-XIPs (cRGD-XIPs-Dox) to B16 melanoma was investigated and compared with XIPs-Dox (non-targeted), cRGD-IPs-Dox (non-crosslinked) and free Dox.

**Results:** cRGD-XIPs were formed with 55.5 wt.% iodine and ca. 90 nm in diameter. cRGD-XIPs-Dox with a Dox loading of 15.3 *wt.*% bared superior colloidal stability and reduction-responsive drug release. Notably, blank cRGD-XIPs showed a maximum-tolerated dose (MTD) > 400 mg iodine equiv./kg while cRGD-XIPs-Dox had an MTD > 150 mg Dox equiv./kg, ca. 15-fold improvement over free Dox. cRGD-XIPs revealed superior CT contrast effect and achieved 46.5- and 24.0-fold better enhancement of CT imaging of B16 melanoma than iohexol at 4 h following intratumoral and intravenous injection, respectively. cRGD-XIPs-Dox displayed an elimination half-life of 6.5 h and an elevated accumulation of 6.68% ID/g in the tumors. Furthermore, cRGD-XIPs-Dox was significantly more effective than XIPs-Dox and cRGD-XPs-Dox in inhibiting growth of B16 melanoma model.

**Conclusion:** This proof-of-concept study demonstrates that cRGD-XIPs are a robust, non-toxic and smart polymeric theranostic agent that can not only significantly enhance CT imaging of tumors but also mediate efficient tumor-targeted chemotherapy. XIPs offer a unique and safe platform for theranostic polymersomes that pre-select patients using CT imaging prior to targeted chemotherapy with the same system.

## 1. Introduction

Theranostics that integrates diagnostics and therapy into a single system provides an emerging treatment modality for diseases like cancers 1-7. X-ray computed tomography (CT) is a frequently applied clinical diagnostic tool 8-12. To enhance CT imaging of soft tissues like tumors in patients, iodine-containing compounds such as iohexol, iodixanol and iopromide are typically used 13-15. In the past years, great interest has put on the development of novel nano-CT contrast agents that are able to overcome the problems of small molecular contrast agents, such as fast elimination, high doses needed, low tumor selectivity, potential renal toxicity, and possible iodine hypersensitivity 16-18, to achieve dual and multi-modal imaging 19-21, and to combine CT with various therapies 22. For example, inorganic nanoparticles based on gold 23, 24, copper chalcogenides 25, transition-metal dichalcogenides 26, rare earth 27, and iodinated organic nanosystems including liposomes 28, nanogels 29, nanoemulsions 30, nanoparticles 31, and dendritic polymers 32 have been investigated for simultaneous tumor CT imaging and therapy. These nanotheranostic systems demonstrate not only enhanced imaging but also potent anti-tumor efficacy. However, despite their excellent X-ray attenuation coefficient, the clinical translation of inorganic nanoparticles is limited by their safety and high cost concerns. While iodinated nanosystems typically possess inadequate CT contrast coefficients, mainly due to low iodine contents. In addition to complicated synthesis process, high viscosity of iodinated dendritic polymers stands as a critical shortcoming against their utilization. In our previous work, nanopolymersomes containing high iodine based on poly(ethylene glycol)-*b*-poly(iodinate trimethylene carbonate) (PEG-*b*-PIC) exhibited significant CT imaging enhancement of the blood pool and malignant tumors in contrast with iohexol 33. Moreover, low *in vivo* stability and slow drug release inside tumor cells are additional problems for nano-CT contrast agents to be applied for therapeutic purposes. We and others verified that disulfide crosslinked nanoparticles show superb *in vivo* stability and accelerated intracellular drug release 34-36.

**Figure 1:**
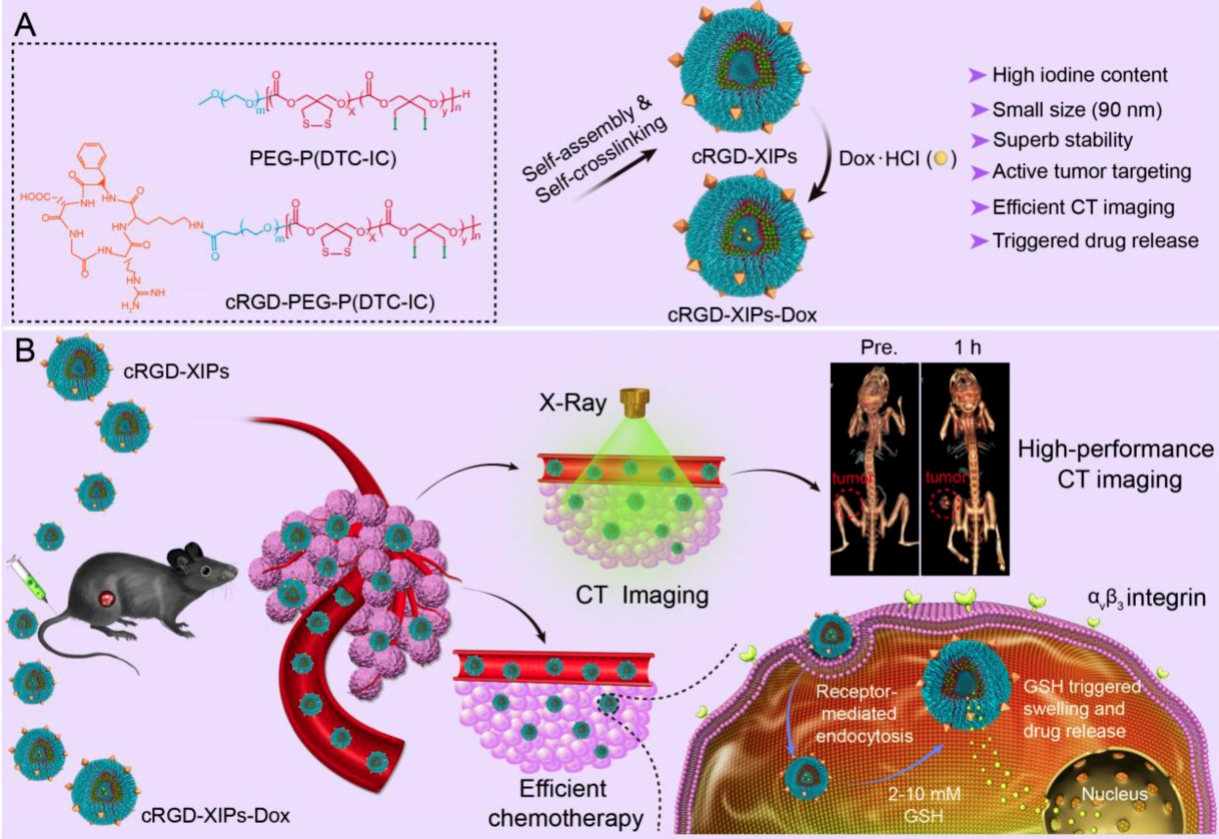
Illustration of the fabrication and properties of cRGD-functionalized disulfide-crosslinked iodine-rich polymersomes (cRGD-XIPs) and Dox-loaded cRGD-XIPs (cRGD-XIPs-Dox) (**A**), and cRGD-XIPs as robust nano-CT contrast agents for enhanced CT imaging of tumor and cRGD-XIPs-Dox as smart nanomedicines for targeted chemotherapy (**B**).

Here, cyclic RGD directed, disulfide crosslinked iodine-rich polymersomes (cRGD-XIPs) were designed based on biodegradable poly(ethylene glycol)-*b*-poly(dithiolane trimethylene carbonate-*co*-iodinated trimethylene carbonate) copolymer (PEG-P(DTC-IC)) and cRGD-PEG-P(DTC-IC) for targeted CT imaging of an α_v_β_3_-integrin positive B16 melanoma model (**Fig. 1**). We further examined the anti-tumor efficacy of doxorubicin-loaded cRGD-XIPs (cRGD-XIPs-Dox) in mice bearing B16 melanoma. cRGD has been extensively explored as an active targeting ligand to α_v_β_3_ integrin over-expressing cancer cells including B16 cells 37-41. It should be noted that even for the same type of tumor, different patients might have vastly different responses to targeted therapies, owing to the differences in tumor physical, pathological and biological properties. The screening of patients is thus of critical importance to targeted nanomedicines 42-44. This study aimed to develop theranostic polymersomes that pre-select patients using CT imaging prior to targeted chemotherapy with the same system. Interestingly, these novel theranostic polymersomes exhibited small size (90 nm), high iodine content of 55.5 *wt.*%, iso-osmolality, high Dox loading content (15.3 *wt.*%), superior colloidal stability, and fast intracellular drug release. cRGD-XIPs could greatly improve the *in vivo* CT imaging via intratumoral or intravenous injection compared with iohexol. Moreover, cRGD-XIPs-Dox was far more potent in retarding the growth of B16 tumor than non-targeted XIPs-Dox and non-crosslinked cRGD-IPs-Dox controls.

## 2. Materials and methods

### 2.1 Synthesis of PEG-P(DTC-IC) and cRGD-PEG-P(DTC-IC) block copolymers

Briefly, under a nitrogen atmosphere, MeO-PEG-OH (*M*_n_ 5.0 kg/mol, 0.1 g, 20 μmol), DTC (0.1 g, 0.52 mmol), IC (1.0 g, 2.62 mmol) and DCM (5.0 mL) was charged into a schlenk bottle and stirred to dissolve using a magnetic stirrer. To the solution, zinc bis[bis(trimethylsilyl)amide] (29 mg, 75 μmol) was quickly added. The ring opening polymerization (ROP) proceeded at 40 °C for 48 h before termination, and the resulting copolymer was precipitated in cold diethyl ether, filtrated and dried in vacuo for 24 h. Yield: 84.2%. Similarly, NHS-PEG-P(DTC-IC) was synthesized using NHS-PEG (*M*_n_ = 6.5 kg/mol) as an initiator. Yield: 82.1%. Then, cyclic cRGDfK (16 mg, 2.62 mmol) was conjugated to NHS-PEG-P(DTC-IC) (0.5 g, 1.31 mmol) yielding cRGD-PEG-P(DTC-IC) similarly as reported 40. Yield: 85.6%. The degree of cRGD conjugation was 92% as determined by micro BCA protein assays. Briefly, 10 µL DMF solution of cRGD-PEG-P(DTC-IC) (10 mg/mL) was diluted to 0.1 mg/mL in water. A 20 μL aliquot was added into a 96-well plate and mixed with 100 μL of working reagent of micro BCA (Reagent A and B = 50/1). The plate was mixed well and kept at 37 °C for 60 min and measured the absorbance at 570 nm using a microplate reader. The absorbance of Mal-PEG-P(DTC-IC) solution prepared similarly was subtracted. The cRGD conjugation was calculated from the standard curve of cRGD peptide of known concentrations.

### 2.2 Preparation of cRGD-XIPs and cRGD-XIPs-Dox

Typically, 900 μL of phosphate buffer (PB, 10 mM, pH 7.4) was added dropwise to 100 μL DMF solution (10.0 mg/mL) of PEG-P(DTC-IC) and cRGD-PEG-P(DTC-IC) (4/1, w/w) under stirring. After 20 min, the mixture was dialyzed against PB for 12 h (Spectra/Pore, MWCO 7000), placed in a shaking-bed at 37 °C for 24 h yielding self-crosslinked polymersomes cRGD-XIPs. The colloidal stability at low concentration (50 mg/L) and in the presence of 10% serum or 10 mM GSH was investigated using DLS.

cRGD-XIPs-Dox was fabricated by loading Dox into preformed cRGD-XIPs via a pH-gradient method. Briefly, 2.7 mL citrate buffer (10 mM, pH 4.0) was dropwise adding into 0.3 mL DMF solution of PEG-P(DTC-IC) and cRGD-PEG-P(DTC-IC) (10 mg/mL). After stirring for 15 min, the pH was adjusted to 8.0 with disodium hydrogen phosphate (4 M). Then, 60, 120, or 180 μL Dox•HCl solution (5 mg/mL), corresponding theoretical DLC of 9.7, 16.7, or 23.1 wt.%, respectively) was added and stirred at 37 °C for 5 h in dark. cRGD-IPs-Dox was obtained via dialysis procedure similar as above. Dox loading content (DLC) and loading efficiency (DLE) was quantified using fluorescence (Agilent Cary Eclipse) measurement (ex. 480 nm and em. 560 nm) 40, 45 using the following formula:

DLC (wt.%) = weight of loaded drug/total weight of polymer and loaded drug) × 100

DLE (%) = (weight of loaded drug/weight of drug in feed) × 100

### 2.3 *In vivo* pharmacokinetics and biodistribution

The mice were handled under protocols approved by Soochow University Laboratory Animal Center and the Animal Care and Use Committee of Soochow University. cRGD-XIPs-Dox, XIPs-Dox, cRGD-IPs-Dox and free Dox (10 mg Dox/kg) in PBS were injected into C57BL/6 mice (18-20 g) via the tail veins (n = 3), and ca. ~50 μL blood was taken from the eye socket of the mice at prescribed time post injection. The blood samples upon withdrawing were mixed with 100 µL Triton X-100 solution (1%) with brief sonication. 500 µL DMF (containing 20 mM DTT) was introduced to extract Dox. The samples were stored at -20 °C overnight, centrifuged, and Dox in the supernatant was determined by fluorometry based on a calibration curve of Dox solution of known concentrations (supporting information). Dox expressed as percent injected dose per gram (%ID/g) was plotted as a function of time. The half-lives of two phases (t_1/2,α_ and t_1/2,β_) and area under the curve (AUC) were derived using Origin 8.

For biodistribution studies in female C57BL/6 mice (18-20 g), 50 μL of B16 cells (1 × 10^6^) were subcutaneously injected in the right hind flank. When the tumors grew to *ca.* 200 mm^3^ after around 10 days, cRGD-XIPs-Dox, XIPs-Dox and free Dox (10 mg Dox/kg, 200 µL) was intravenously (*iv*) injected via tail veins (n = 3). After 4 h, the tumors and major organs were excised, washed, weighed, and homogenized in 500 µL Triton X-100 (1%). Subsequently, 1 mL DMF (containing 20 mM DTT) was added to extract Dox, and the samples were treated as above based on calibration curves of Dox of known concentrations in different tissue individually (supporting information).

### 2.4 Maximum-tolerated dose (MTD) determination

The healthy C57BL/6 mice were randomly divided into 6 groups (n = 3) and a single dose of cRGD-XIPs (300 or 400 mg I equiv./kg), cRGD-XIPs-Dox (100 or 150 Dox/kg), or free Dox (5 or 10 mg Dox/kg) were *iv* injected. Body weight, behavior and survival of mice were examined for 10 days. MTD was defined as the dose that causes neither mouse death due to the toxicity, nor >15% of body weight loss, nor other remarkable changes in the general appearance within 10 days.

### 2.5 CT imaging of mice with intratumoral (it) and intravenous (iv) injection of cRGD-XIPs

B16 tumor-bearing C57BL/6 mice were used as a α_v_β_3_ integrin over-expressing tumor model to evaluate the targeted CT contrast enhancement of tumors *in vivo*. The mice were randomly divided into two groups (n = 3), and treated with cRGD-XIPs and iohexol at 350 mg I equiv./kg (50 μL) via *it* injection. Before and 5 min, 1 h and 4 h after *it* injection, the CT scan was carried out using a GE discovery CT750 HD (GE Healthcare, WI) setting parameters as follows: beam collimation = 64 ×0.625 mm; table speed = 27 mm per rotation; beam pitch = 1.25; gantry rotation time = 1.0 s.

The tumor bearing mice were also* iv* injected with cRGD-XIPs, XIPs or iohexol (350 mg I equiv./kg) in 200 μL PB. The CT images were obtained before and 40 min, 2 h, 4 h and 6 h post *iv* injection as described above.

### 2.6 *In vivo* antitumor efficacy of cRGD-XIPs-Dox

The female B16 tumor-bearing mice (tumor volume: 50-100 mm^3^) were randomly divided into six groups (n = 5): cRGD-XIPs-Dox (5 or 10 mg Dox/kg), XIPs-Dox (10 mg Dox/kg), cRGD-IPs-Dox (10 mg Dox/kg), free Dox (5 or 10 mg Dox/kg), and PBS. The formulations were* iv* injected every 2 days. The first day of the injection was labeled as day 0. The tumor size was measured every two days and the volume was calculated according to the formula V = 0.5 × L ×W^2^, wherein L and W are the tumor dimension at the longest and widest point, respectively. Mice were weighed and normalized to the initial weights. On day 10, the tumors were excised, weighed and photographed. Heart, liver, spleen, lung and kidney were sliced and prepared for H&E and then observed with a digital microscope (Leica QWin, Germany). The tumor inhibition rate (TIR) was calculated:

TIR (%) = (1- tumor weight of treatment group/ tumor weight of PBS group) ×100.

### 2.7 Statistics

All data are presented as the mean ± standard deviation (SD). One-way analysis of variance (ANOVA) was used to determine significance among groups, after which post-hoc tests with the Bonferroni correction were used for comparison between individual groups. Statistical significance was established at *p < 0.05, **p < 0.01 and ***p < 0.001.

## 3. Results and discussion

### 3.1 Fabrication of cRGD-XIPs and cRGD-XIPs-Dox

cRGD-XIPs were co-self-assembled from PEG-P(DTC-IC) and cRGD-PEG-P(DTC-IC). The ROP of IC and DTC monomers using MeO-PEG-OH (*M*_n_ = 5.0 kg/mol) as a macro-initiator afforded PEG-P(DTC-IC) (**Fig. S1**), similar to our previous report 33. ^1^H NMR analyses (**Fig. S2**) revealed that the P(DTC-IC) block had an *M*_n_ of 52.9 kg/mol and DTC/IC weight ratio of 8.7/91.3 (**Table S1**). GPC measurement revealed that the molecular weight distribution of PEG-P(DTC-IC) was unimodal (*M*_w_/*M*_n_ = 1.31), and *M*_n_ was 63.2 kg/mol, close to that determined by ^1^H NMR (57.9 kg/mol) and the theoretical value (60.0 kg/mol) (**Table S1**), endorsing controlled synthesis of PEG-P(DTC-IC). Likewise, NHS-PEG-P(DTC-IC) was obtained with similar P(DTC-IC) block molecular weight (*M*_n_ = 53.9 kg/mol) and composition (DTC/IC = 8.7/91.3, w/w) by copolymerization of DTC and IC initiated by NHS-PEG-OH (*M*_n_ = 6.5 kg/mol) (**Fig. S3**). The conjugation of cRGDfK to NHS-PEG-P(DTC-IC) by carbodiimide chemistry yielded cRGD-PEG-P(DTC-IC). Micro BCA protein assay results showed a cRGD functionality of 92% (**Fig. S4**). Notably, the molecular weight of PEG in cRGD-PEG-P(DTC-IC) was longer than that in PEG-P(DTC-IC) (6.5 *vs.* 5.0 kg/mol) to achieve better exposure of cRGD for optimal targeting. Differential scanning calorimetry (DSC) measurements confirmed the amorphous structure of PEG-P(DTC-IC) and cRGD-PEG-P(DTC-IC) with glass transition temperatures (*T*_g_) of -25.3 and -21.5 ℃, respectively (**Table S1**), indicating that they are rubbery and flexible at body temperature.

**Figure 2:**
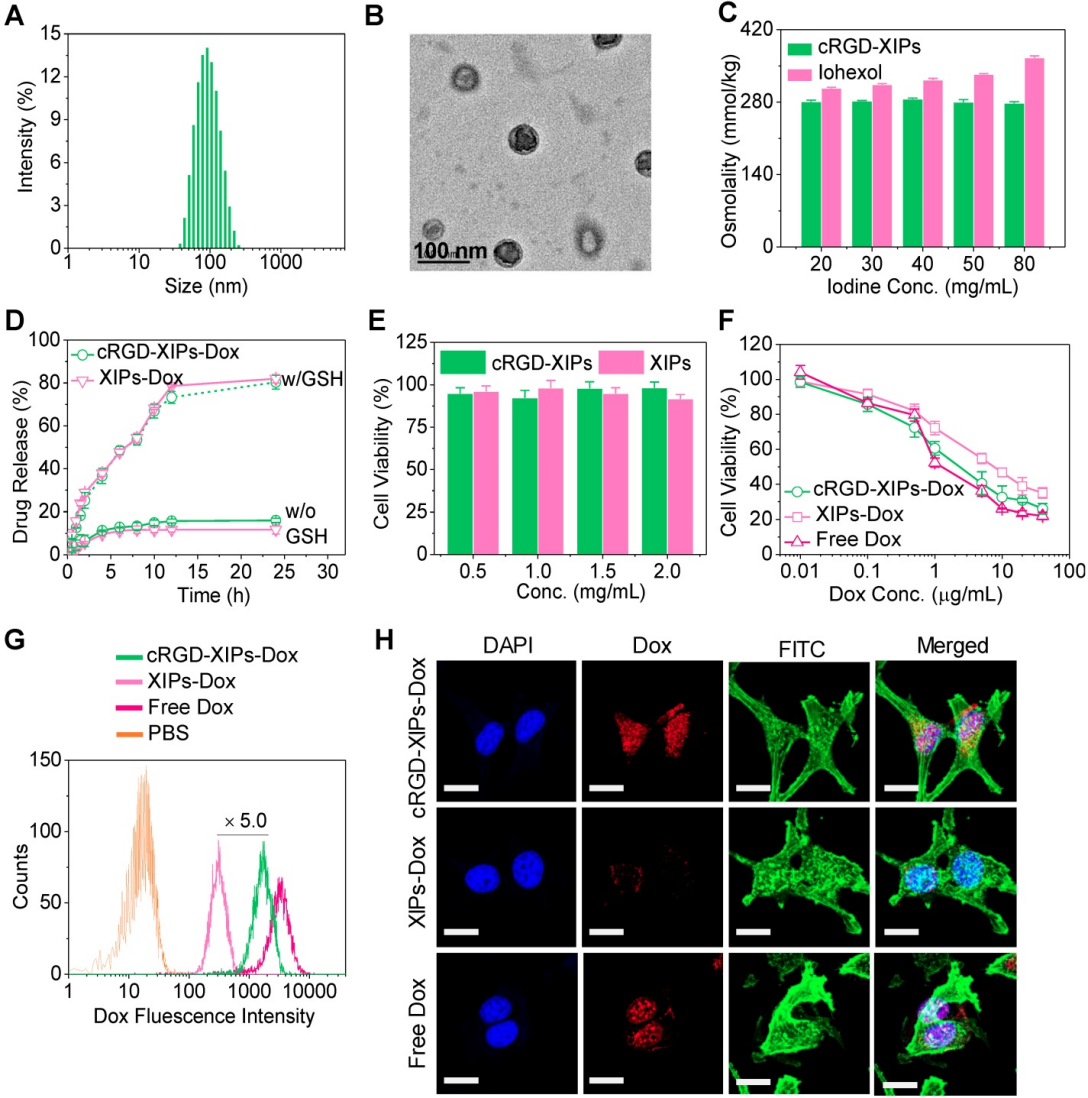
Characterizations of cRGD-XIPs and cRGD-XIPs-Dox. Size distribution (**A**), TEM image (**B**), and osmolality as a function of concentration (iohexol as control) (**C**) of cRGD-XIPs. (**D**) *In vitro* Dox release study of cRGD-XIPs-Dox and XIPs-Dox in PB with or without 10 mM GSH at 37 °C. Polymersomes concentration was 50 mg/L. (**E**) Cytotoxicity of empty XIPs and cRGD-XIPs to B16 cells following 48 h incubation. (**F**) Antitumor activity of cRGD-XIPs-Dox to B16 cells. The cells following 4 h incubation with Dox formulations were further cultured for 44 h. Flow cytometric analyses (**G**) and CLSM observation (**H**) of B16 cells following 4 h incubation with cRGD-XIPs-Dox **(**Dox: 10.0 μg/mL). The images from left to right are cell nuclei stained by DAPI (blue), Dox (red), cytoskeleton stained by phalloidin-FITC (green), and the merged images. Scale bars: 20 μm. XIPs-Dox and free Dox were used as controls in **F, G, H.**

The co-self-assembly of PEG-P(DTC-IC) and cRGD-PEG-P(DTC-IC) (w/w, 8/2) in water yielded cRGD-XIPs of ca. 90 nm in diameter and a size distribution (PDI) of 0.10 (**Fig. 2A**) via a solvent exchange method. The addition of PB into a DMF solution of amphiphilic block copolymers increased the interfacial tension between P(DTC-IC) segment and water, rendering P(DTC-IC) segment insoluble thus triggering copolymer self-assembly into polymersomes. A cRGD density of 20% in polymersomes was investigated as nanoparticles of this density displayed the best targetability 36, 38. TEM micrograph verified a spherical hollow structure (**Fig. 2B**). UV-Vis measurements displayed that the characteristic absorbance of dithiolane ring at 320 nm disappeared after polymersome work-up procedure (**Fig. S5**), revealing the spontaneous dithiolanes crosslinking in the polymersome membrane. As a result, no critical aggregation concentration (CAC) could be detected. Interestingly, cRGD-XIPs displayed excellent colloidal stability during two-month storage at room temperature and against dilution or in the presence of 10% serum for 48 h (**Fig. S6**), due to the spontaneous disulfide-crosslinking of the membrane as reported for other DTC-containing polymersomes 35, 40. Accordingly, cRGD-XIPs could undergo fast destabilization in 10 mM GSH as determined by both DLS and TEM measurements (**Fig. S7)**, owing to disulfide cleavage and de-crosslinking. Notably, cRGD-XIPs exhibited an iso-osmotic pressure of 280 mmol kg^-1^ at iodine contents ranging from 20 to 80 mg I/mL (**Fig. 2C**), while the osmolality of iohexol increased with concentration, reaching 366 mmol kg^-1^ at 80 mg I/mL. XIPs self-assembled from PEG-P(DTC-IC) only displayed almost the same biophysical properties and were used as a non-targeted control.

Doxorubicin hydrochloride (Dox) could be efficiently loaded into cRGD-XIPs and XIPs via pH-gradient method, in which the pH values of inner and outer of the polymersomes were 4.0 and 8.0, respectively. The drug loading content (DLC) increased with increasing theoretical DLC and cRGD-XIPs-Dox achieved a high DLC of 15.3 *wt.*%. cRGD-XIPs-Dox enlarged from 80, 84, to 92 nm with increasing DLC from 6.6, 12.0, to 15.3 wt.% (**Table 1**). Notably, all cRGD-XIPs-Dox presented a low PDI (0.06-0.10) and close to neutral surface charge. XIPs-Dox had nearly identical size distribution, drug loading, and zeta potential to cRGD-XIPs-Dox (**Table 1**), indicating little influence of cRGD peptide on polymersomal Dox. **Fig. 2D** shows that at a polymersome concentration of 50 mg/L, Dox release from XIPs-Dox and cRGD-XIPs-Dox was less than 16% in 24 h. Importantly, under an intracellular mimicking condition (10 mM GSH), cRGD-XIPs-Dox dumped more than 80% Dox within 24 h (**Fig. 2D**). In contrast, within 12 h over 40% Dox leaked out of the non-crosslinked counterparts, IPs-Dox and cRGD-IPs-Dox, which were based on PEG-PIC and cRGD-PEG-PIC/PEG-PIC, respectively (**Fig. S8**). The results demonstrate the important role of disulfide crosslinking in preventing drug leakage at physiological condition and in triggering fast drug release intracellularly.

### 3.2. *In vitro* cytotoxicity and targetability of cRGD-XIPs-Dox

cRGD is widely used as a targeting motif toward α_v_β_3_ integrin over-expressing tumor cells including B16 melanoma cells 46, 47. Here, we evaluated the cytotoxicity of blank cRGD-XIPs and cRGD-XIPs-Dox in B16 cell model using MTT assays. **Fig. 2E** shows that XIPs and cRGD-XIPs had no toxicity at concentrations ≤ 2.0 mg/mL, as reported for non-crosslinked polymersomes 33, confirming their excellent biocompatibility. In comparison, cRGD-XIPs-Dox showed high potency to B16 cells with an IC_50_ (half-maximal inhibitory concentration) of 2.33 μg Dox/mL, which was comparable to free Dox and 3.3-fold lower than XIPs-Dox (**Fig. 2F**).

Flow cytometric analyses displayed that cRGD-XIPs-Dox reveal ca. 5.0-fold higher cellular uptake by B16 cells than XIPs-Dox following 4 h incubation (**Fig. 2G**). Interestingly, both cRGD-XIPs-Dox and XIPs-Dox had low cellular uptake by L929 fibroblasts, further verifying their specific targetability (**Fig. S9**). CLSM images showed that Dox fluorescence in the nuclei of cells treated with cRGD-XIPs-Dox accumulated gradually from 0.5 h to 2 h and mostly in the nuclei at 4 h, which was considerably more intensive than those of XIPs-Dox treated cells and free cRGD pretreated B16 cells (**Fig. 2H & Fig. S10**). The above results corroborate that cRGD-XIPs-Dox can be efficiently internalized by B16 cells via receptor-mediated endocytosis mechanism 48, 49, and Dox releases quickly intracellularly.

### 3.3. Pharmacokinetics, biodistribution and safety *in vivo*

**Figure 3:**
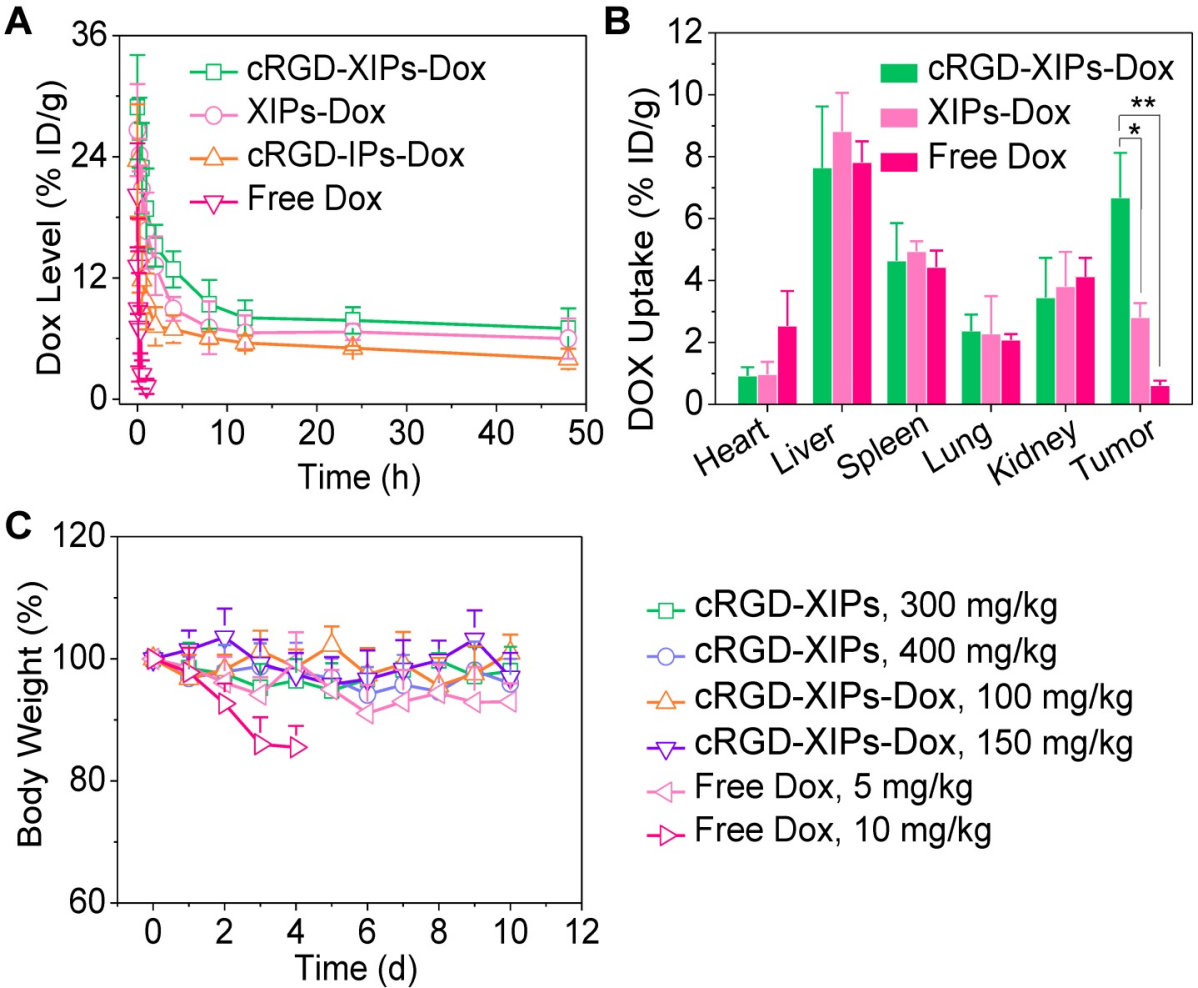
*In vivo* pharmacokinetics, biodistribution and toleration studies of cRGD-XIPs-Dox in mice (n = 3). (**A)** Pharmacokinetics of cRGD-XIPs-Dox in healthy C57BL/6 mice (10 mg Dox/kg). XIPs-Dox (non-targeted), cRGD-IPs-Dox (non-crosslinked) and free Dox were used as controls. (**B**) Biodistribution of free Dox and Dox delivered by cRGD-XIPs or XIPs in C57BL/6 mice bearing B16 tumor 4 h post* iv* injection. (**C**) Toleration studies of tumor-free C57BL/6 mice towards cRGD-XIPs, cRGD-XIPs-Dox and free Dox. *p< 0.05 and **p < 0.01 (one-way Anova and Tukey multiple comparisons tests).

The pharmacokinetics studies demonstrated that the blood circulation followed a typical two-compartment model: a rapid decline in distribution phase and long period elimination phase, and the circulation of Dox was significantly extended by loading into cRGD-XIPs and XIPs as calculated from the calibration curve of Dox of known concentrations in the presence of blood (**Table S2**). cRGD-XIPs-Dox and XIPs-Dox showed an elimination half-life (t_1/2,β_) of about 6.5 h, which was ca. 50- and 1.8-fold longer than free Dox (0.13 h) and cRGD-IPs-Dox (non-crosslinked control, 3.61 h), respectively (**Fig. 3A**). Notably, commercial contrast agent iohexol has a short t_1/2,β_ of 24.6 min 50, 51. The AUC (area under the curve) of cRGD-XIPs-Dox was 97.76 h‧µg/mL, which was 1.8- and 26.4-time bigger than that of cRGD-IPs-Dox and free Dox, respectively.

The quantitative biodistribution of Dox in cRGD-XIPs-Dox treated mice 4 h following *iv* injection was determined from calibration curves of Dox in the presence of individual tissue (**Table S2**), and demonstrated that Dox accumulation in the tumors was 6.68% of injected dose per gram of tissue (% ID/g). The accumulation was ca. 2.4- and 11.0-fold higher than that of XIPs-Dox (2.81% ID/g) and free Dox (0.61% ID/g), respectively (**Fig. 3B**). **Table S3** shows clearly that cRGD-XIPs-Dox had higher tumor-to-normal tissue (T/N) ratios in all major organs than free Dox and XIPs-Dox. Notably, cRGD-XIPs-Dox treated mice showed T/N ratios of Dox in the heart was ca. 30 times higher than free Dox treated ones, signifying the possibly significant reduction of cardiotoxicity, which is a dose-limiting side effect for Dox 52, 53.

To explore their theranostic window, we assessed the safety of blank cRGD-XIPs and cRGD-XIPs-Dox in C57BL/6 tumor-free mice. Remarkably, a single dose of cRGD-XIPs at 300 and 400 mg I equiv./kg, or cRGD-XIPs-Dox at 100 and 150 mg Dox/kg did not provoke obvious loss in body weight or change of behavior of the mice within the experiment period of 10 days (**Fig. 3C**). In contrast, dramatic body weight loss was caused by free Dox at 10 mg/kg, indicating over 15-fold better toleration of cRGD-XIPs-Dox than free Dox. Safety profile is one of the critical requirements for clinical translation of theranostic agents 54, 55. This high MTD of cRGD-XIPs-Dox was ascribed to the excellent biocompatibility of the materials, reduced non-specific uptake by normal organs resulting from cRGD active targeting effect, and high stability preventing Dox leakage and damage of the normal tissues from the crosslinked polymersomal structure.

### 3.4. CT imaging performance of cRGD-XIPs *in vivo*

**Figure 4:**
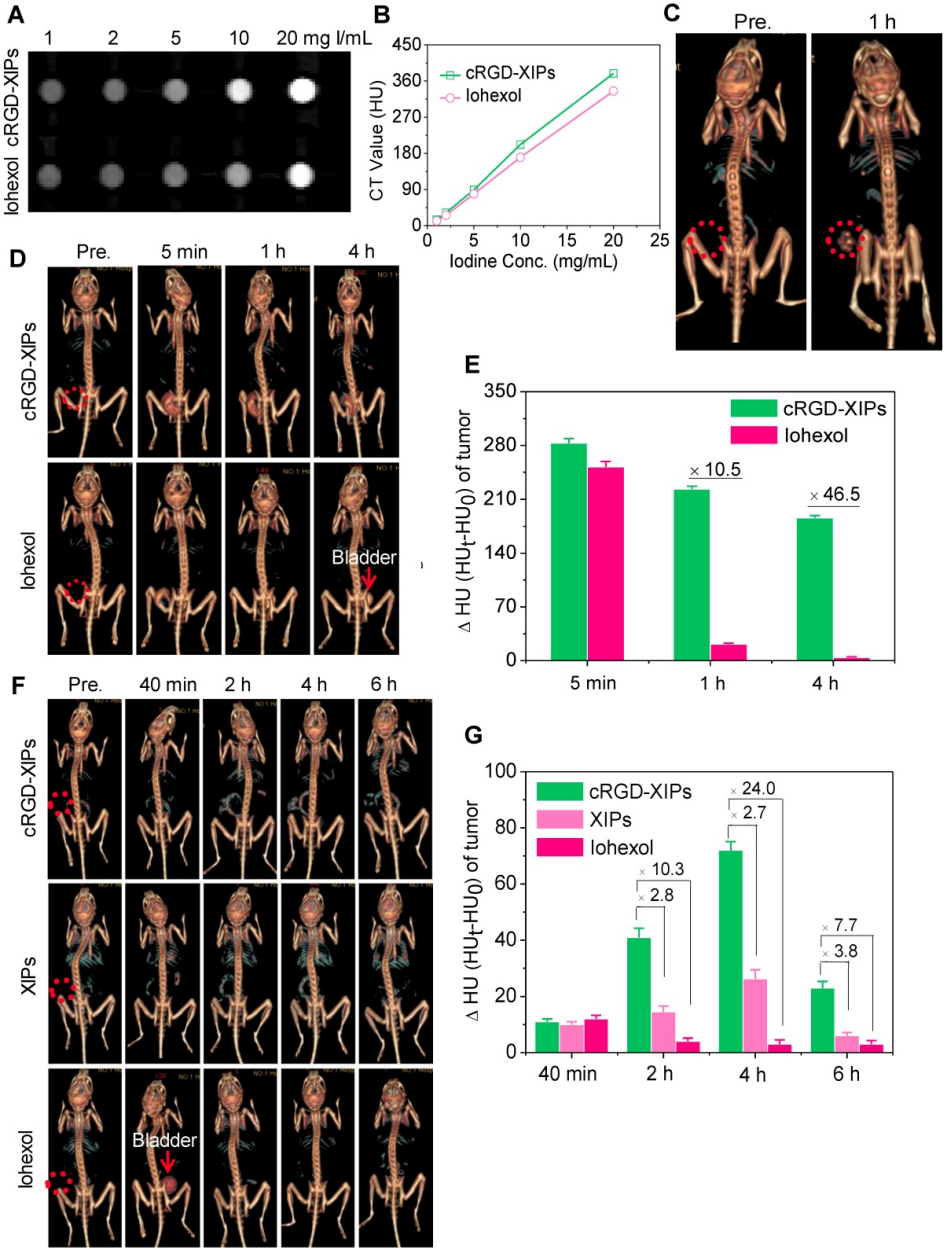
CT images (**A**) and X-ray attenuation (**B**) of iohexol and cRGD-XIPs solutions as a function of iodine concentrations. (**C**) 3D reconstruction CT images of tumors in mice before and 1 h after *it* administration of cRGD-XIPs or iohexol. 3D reconstruction CT images (**D**) and enhanced CT attenuation (ΔHU) (**E**) of the tumors before and at 5 min, 1 h or 4 h after *it* injection of cRGD-XIPs or iohexol. 3D reconstruction CT images (**F**) and ΔHU (**G**) of the tumors before and at different time post *iv* injection of cRGD-XIPs, XIPs or iohexol. The red dotted circles in** B, D** and **F** indicate the tumor areas. The iodine dose was set at 350 mg I equiv./kg for **C-G**.

Despite its wide use in the clinics, CT imaging of soft tissues like tumors is challenging. To improve the CT imaging contrast, nano-contrast agents containing elements of high atomic numbers (e.g. iodine, gold) have been explored 56, 57. Here, we investigated cRGD-XIPs for CT imaging of subcutaneous B16 melanoma as a model tumor. Melanoma is a very aggressive type of skin cancer and has high risk of lymphatic metastasis 58, 59. **Fig. 4A** displays that cRGD-XIPs presented good CT contrast. The calculated CT attenuation as a function of iodine concentrations (measured in Hounsfield units (HU) revealed that cRGD-XIPs had even better attenuation coefficient than iohexol (**Fig. 4B**).

For* in vivo* CT imaging, 50 μL of cRGD-XIPs or iohexol (350 mg I equiv./kg) was intratumorally (*it*) administrated into C57BL/6 mice bearing B16 tumors, and then scanned by CT. At 1 h after injection, CT images of cRGD-XIPs treated mice showed strong tumor contrast, and the CT attenuation increased from 35.4 ± 4.8 HU before injection to 221.9 ± 8.7 HU (**Fig. 4C**). The CT images acquired at different time intervals following* it* injection further showed that strong CT signals were observed in the tumors from 5 min to 4 h post *it* injection of cRGD-XIPs (**Fig. 4D**). In comparison, from 1 h post *it* injection of iohexol, no CT contrast could be discerned. **Fig. 4E** displays that the tumors of cRGD-XIPs group had about 10.5- and 46.5-fold higher ΔHU than iohexol group at 1 and 4 h post-injection, respectively. It is noted that very sharp CT signals could be detected in the bladder of iohexol group, which accorded well with the fast renal clearance of small molecular contrast agents 60, 61. The results of *it* injection illustrate the clear benefit of utilizing stable cRGD-XIPs over iohexol for prolonged retention in the region of interest for CT imaging.

We further studied the CT imaging of B16 tumors in mice following intravenous (*iv*) injection of cRGD-XIPs, XIPs and iohexol (350 mg I equiv./kg). **Fig. 4F** shows that cRGD-XIPs afforded the best CT images of the tumors in 3D reconstruction mode or axial mode. The quantification of CT attenuation revealed that ΔHU in the tumors of cRGD-XIPs group increased from 40 min to 4 h (**Fig. 4G**). Notably, cRGD-XIPs gave 2.7- and 24.0-fold higher contrast than XIPs and iohexol at 4 h, respectively. It is interesting to note that cRGD-XIPs did not show a high CT signal in liver (**Fig. 4F** & **Fig. S11**), in contrast to cRGD-XIPs-Dox that revealed a high liver accumulation besides a high tumor accumulation (**Fig. 3B**). The difference between biodistribution and CT imaging results most probably comes from liver saturation effect, *i.e.* liver uptake of cRGD-XIPs was saturated above certain level. CT imaging was performed at over 7.7-fold higher cRGD-XIPs dosage than for biodistribution studies. The high tumor contrast and low liver contrast of cRGD-XIPs renders CT imaging a particularly interesting tool for validating their tumor-targetability.

### 3.5. The anti-tumor activity of cRGD-XIPs-Dox in mice bearing B16 tumor

**Figure 5:**
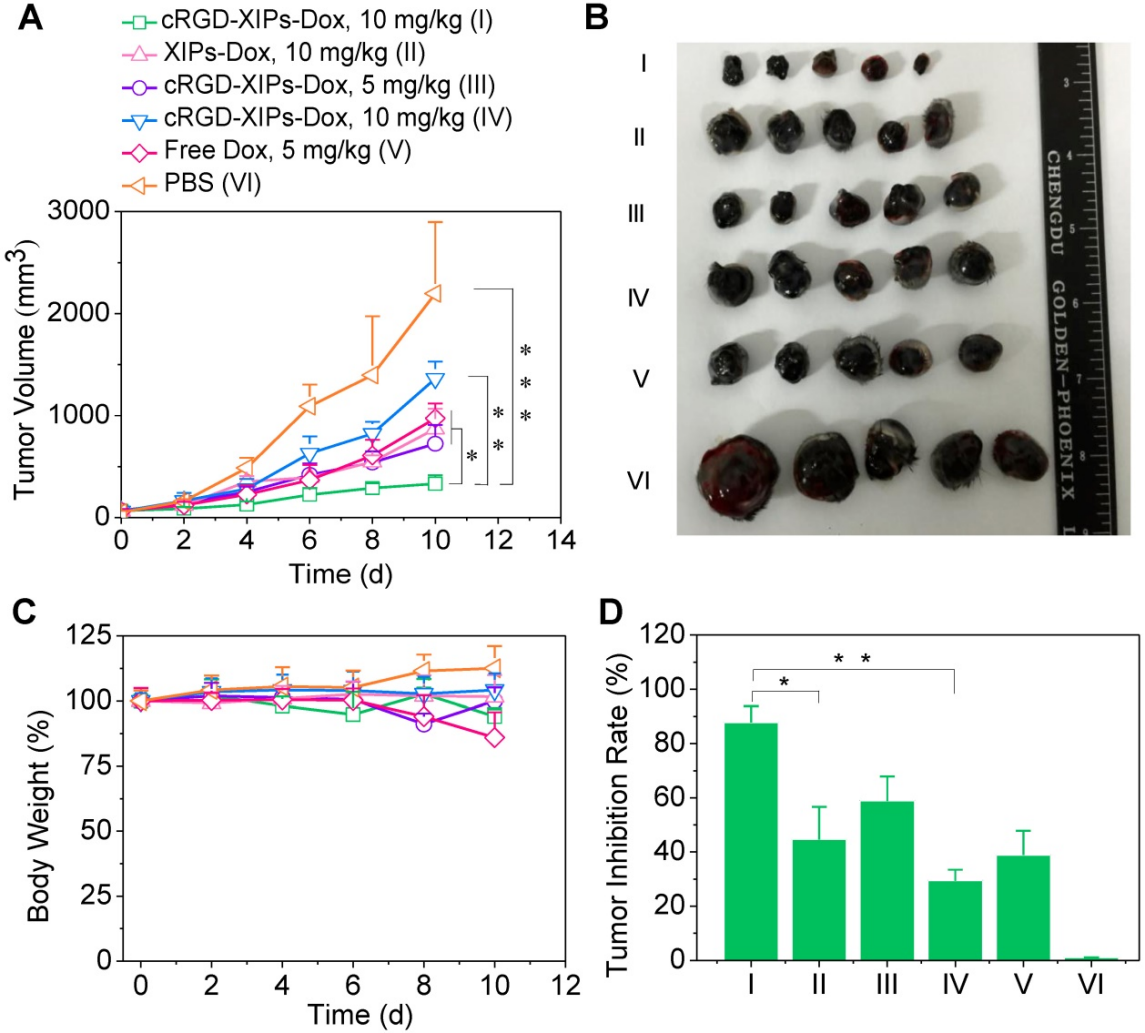
*In vivo* evaluation of the therapeutic efficacy of cRGD-XIPs-Dox using B16 tumor-bearing mice. XIPs-Dox, cRGD-IPs-Dox, free Dox and PBS were used as controls. The drugs were administrated on day 0, 2, 4, 6 and 8 (5 or 10 mg Dox/kg). (**A**) Tumor volume changes. (**B**) Photographs of tumors collected on day 10. (**C**) Body weight changes. (**D**) Tumor inhibition rates on day 10. Data are presented as mean ± SD (n = 5). *p< 0.05, **p < 0.01, and ***p < 0.001 (one-way Anova and Tukey multiple comparisons tests).

With validated targetability of cRGD-XIPs to α_v_β_3_-integrin over-expressing B16 melanoma by CT imaging, the* in vivo* antitumor activity of cRGD-XIPs-Dox to B16 tumor was then studied and compared with XIPs-Dox (non-targeted), cRGD-IPs-Dox (non-crosslinked) and free Dox formulation. B16 tumor was highly aggressive and grew from 52 mm^3^ to 2197.0 mm^3^ in 10 days (PBS group, **Fig. 5A**). Notably, cRGD-XIPs-Dox at 10 mg Dox/kg displayed the best tumor inhibition, being significantly more effective than XIPs-Dox and cRGD-IPs-Dox controls at the same dosage, certifying the pivotal roles of both cRGD targeting and disulfide crosslinking. In consideration of its dose-limiting effect, free Dox was given at 5 mg/kg. The results showed that free Dox was less potent in tumor inhibiting than cRGD-XIPs-Dox at 5 mg Dox/kg. The photographs of tumors collected on day 10 were consistent with tumor volumes (**Fig. 5B**). Moreover, cRGD-XIPs-Dox, XIPs-Dox and cRGD-IPs-Dox treated mice showed little body weight loss (**Fig. 5C**), in collaborating with the high MTD of these polymersomal formulations. **Fig. 5D** reveals a remarkable tumor inhibition rate (TIR) of 87.8% for cRGD-XIPs-Dox at 10 mg Dox/kg, and this was significantly higher than other groups. H&E staining showed that cRGD-XIPs-Dox did not induce significant damage to major organs (**Fig. S12**). Free Dox, however, induced obvious damage of liver, kidney and heart. Hence, despite of slightly better tumor suppression ability of cRGD-XIPs-Dox at 5 mg/kg than free Dox, further increase of the dose to 10 mg/kg produced excellent tumor inhibition with little adverse effects. cRGD-XIPs-Dox can thus be used as a safe and efficient therapeutic agent showing not only improved safety but also active targetability and better therapeutic efficacy toward B16 tumor in mice.

## Conclusion

We have demonstrated that cRGD-functionalized, disulfide-crosslinked, biodegradable iodine-rich polymersomes (cRGD-XIPs) bare small size, low toxicity and superior CT imaging of α_v_β_3_ integrin overexpressed tumors to clinical agent iohexol *in vivo*. Importantly, cRGD-XIPs can efficiently load Dox. Dox-loaded cRGD-XIPs (cRGD-XIPs-Dox) possess several appealing features like high stability, reduction-triggered drug release, prolonged circulation time and active targetability to B16 melanoma, resulting in elevated tumor accumulation and significantly more effective suppression of B16 melanoma than XIPs-Dox (non-targeted), cRGD-IPs-Dox (non-crosslinked) and free Dox controls. This proof-of-concept study reveals that cRGD-XIPs are a robust, non-toxic and smart polymer theranostic agent that can not only significantly enhance CT imaging of α_v_β_3_ integrin overexpressing tumors but also mediate efficient tumor-targeted chemotherapy. XIPs offer a unique and safe platform for theranostic polymersomes that pre-select patients using CT imaging prior to targeted chemotherapy with the same system.

## Supplementary Material

Supplementary figures and tables.

## Figures and Tables

**Table 1:** Characterizations of cRGD-XIPs-Dox and XIPs-Dox

Polymersomes	TLC^a^ (wt.%)	DLC^b^ (wt.%)	DLE^b^ (%)	Size^c^ (nm)	PDI^c^	Zeta potential (mV)^ c^
cRGD-XIPs-Dox	9.1	6.6	71.2	80	0.10	+ 0.3
	16.7	12.0	68.3	84	0.06	+ 1.6
	23.1	15.3	60.3	92	0.10	+ 0.9
XIPs-Dox	9.1	6.6	70.4	82	0.09	+ 1.2
	16.7	11.8	66.9	87	0.11	+ 0.5
	23.1	15.2	59.8	89	0.07	+ 0.8

^a^ Theoretical drug loading content. ^b^ Determined using UV spectrometer. ^c^ Determined with a Zetasizer Nano ZS equipped with a DLS and an electrophoresis cell in PB at 1 mg/mL.

## Abbreviations

cRGD: cyclic RGD
CT: computed tomography
PEG-P(DTC-IC): poly(ethylene glycol)-*b*-poly (dithiolane trimethylene carbonate-*co*-iodinated carbonate)
cRGD-PEG-P(DTC-IC): cRGD-poly 76(ethylene glycol)-*b*-poly(dithiolane trimethylene carbonate-*co*-iodinated carbonate)
PEG-PIC: poly(ethylene glycol)-*b*-poly(iodinated carbonate)
DLS: dynamic light scanning
Dox: doxorubicin hydrochloride
DSC: differential scanning calorimetry
TEM: transmission electron microscopy
DTT: dithiothreitol
GSH: glutathione
MTD: maximum tolerated dosage
RES: reticulaendothelial system
MTT: 3-(4,5-dimethylthiazol-2-yl)-2,5-diphenyl tetrazoliumbromide
PBS: phosphate buffer saline
DAPI: 4,6-diamidino-2-phenylindole
CLSM: confocal laser scanning microscopy
T/N: tumor-to-normal tissue
H&E: hematoxylin-eosin staining
HU: Hounsfield units
