# Supplementary material for: Cyclic RGD-Functionalized and Disulfide-Crosslinked Iodine-Rich Polymersomes as a Robust and Smart Theranostic Agent for Targeted CT Imaging and Chemotherapy of Tumor
Source: Theranostics. 2019 Oct 17;9(26):8061–72. doi: 10.7150/thno.37184 (PMC6857068; doi:10.7150/thno.37184)
Supplement: Supplementary file 1 — Supplementary figures and tables. [file thnov09p8061s1.pdf]

Supplementary materials for

Cyclic RGD-Functionalized and Disulfide-Crosslinked Iodine-Rich Polymersomes as a Robust and Smart Theranostic Agent for Targeted CT Imaging and Chemotherapy of Tumor

Yan Zou[1,2,#], Yaohua Wei[1,3,#], Yinping Sun[1], Jie Bao[4], Feirong Yao[4], Zekun Li[1], Fenghua Meng[1,*], Chunhong Hu[4], Gert Storm[3], and Zhiyuan Zhong[1,*]

[1]Biomedical Polymers Laboratory, and Jiangsu Key Laboratory of Advanced Functional Polymer Design and Application, College of Chemistry, Chemical Engineering and Materials Science, and State Key Laboratory of Radiation Medicine and Protection, Soochow University, Suzhou, 215123, P. R. China

[2]International Joint Centre for Biomedical Innovation, School of Life Sciences, Henan University, Jin Ming Avenue, Kaifeng, Henan, 475004, P. R. China

[3]Department of Targeted Therapeutics, MIRA Institute for Biological Technology and Technical Medicine, University of Twente, PO Box 217, 7500AE, Enschede, The Netherlands

[4]Imaging Center, the First Affiliated Hospital of Soochow University, Suzhou, Jiangsu, 215006, P.R. China

[#] These authors contributed equally to this work

*Corresponding authors. Tel/Fax: +86-512-65880098, Email: fhmeng@suda.edu.cn (F. Meng); zyzhong@suda.edu.cn (Z. Zhong)

Materials and methods

Materials

Methoxy poly(ethylene glycol) (MeO-PEG-OH, M_n = 5.0 kg/mol, Đ =1.03, Fluka) and N-hydroxysuccinimide activated poly(ethylene glycol) (NHS-PEG-OH, M_n=6.5 kg/mol, Đ = 1.04, Suzhou Nord Derivatives Pharm-tech Co., Ltd) were dried by azeotropic distillation form anhydrous toluene. Toluene and dichloromethane (DCM) were dried by refluxing over CaH_2 and distilled prior to use. Zinc bis[bis(trimethylsilyl) amide] (97%, Aldrich), cyclic peptide c(RGDfK) (cRGD, 98%, China Peptides Co., Ltd.), iohexol (98%, J&K). Dithiolane trimethylene carbonate (DTC) and iodinated trimethylene carbonate (IC) were synthesized as our previous report [1-2].

Characterizations

The chemical structure of polymers was characterized using ^{1}H NMR on a Unity Inova 400 spectrometer operating at 400 MHz using deuterated chloroform ($CDCl_3$) as a solvent. Gel permeation chromatograph (GPC) measurements were conducted on a Waters 1515 instrument containing two linear PLgel columns (500 Å and Mixed-C), a guard column and a differential refractive-index detector with DMF as an eluent (flow rate: 1.0 mL/min, 30 °C, polystyrene standards). The thermal properties of the copolymers were determined by differential scanning calorimetry (DSC, Q20, TA). The samples were heated from 80 °C to 120 °C at a rate of 20 °C /min, kept at 120 °C for 2 min, cooled to -80 °C at a rate of 300 °C /min, kept at -80 °C for 2 min, and then a second heating scan from -80 °C to 120 °C at a rate of 20 °C /min was recorded. The second heating curves were used to determine the T_m and T_g.

The size, size distribution and zeta potential of polymersomes were determined at 25 °C

using a Zetasizer Nano ZS (Malvern Instruments) equipped with a dynamic light scattering (DLS, 10 mW He-Ne laser, 633 nm wavelength) using back-scattering detection and an electrophoresis cell in PB at 1 mg/mL. Transmission electron microscopy (TEM) images were obtained on a Tecnai G220 TEM (accelerating voltage: 120 kV) with samples prepared by dropping 10 μL of 0.2 mg/mL polymersome suspension on the copper grid and stained by 1 wt.% phosphotungstic acid. The osmolality of iodinated polymersomes cRGD-XIPs and iohexol in PBS (10 mM, pH 7.4) was determined by vapor pressure osometer (WESCOR Inc). The Dox was quantified via fluorescence spectrophotometer (Thermo Scientific, USA) and multi-function microplate reader (VARIOSKAN LUX, Thermo scientific, Ex 480 nm, Em 580 nm). The CLSM images of cells were taken on a confocal laser scanning microscope (TCS SP5, Leica Microsystems CMS GmbH, Germany). Flow cytometric analyses were done using a BD FACS Calibur flow cytometer (Becton Dickinson, USA). The CT scan was performed on a GE discovery CT750 HD (GE Healthcare, WI).

The in vitro drug release from cRGD-XIPs-Dox

The Dox release from XIPs-Dox and cRGD-XIPs-Dox was conducted at a low polymersomes concentration of 50 mg/L using a dialysis tube (Spectra/Pore, MWCO 12000~14000) at 37 °C in two different media, i.e. PB (10 mM, pH 7.4) and PB (10 mM, pH 7.4) containing 10 mM GSH. In order to acquire sink condition, 0.6 mL of XIPs-Dox or cRGD-XIPs-Dox dispersion was dialyzed against 25 mL corresponding medium. At desired time intervals, 5.0 mL of release medium was taken out and replenished with an equal volume of fresh medium. The amount of Dox released in the medium was determined by fluorescence

measurement. The release experiments were conducted in triplicate, and the results presented were the average data with standard deviations.

In vitro cytotoxicity assays

The cytotoxicity of cRGD-XIPs-Dox was determined using B16 melanoma cell line (B16 cells) that expresses a high level of $\alpha_v\beta_3$ integrin. The cells were plated in a 96-well plate (3×10^4 cells/well) using RPMI 1640 media supplemented with 10% fetal bovine serum, 1% L-glutamine, and antibiotics penicillin (100 IU/mL) and streptomycin (100 μg/mL). After 24 h, cRGD-XIPs-Dox, XIPs-Dox and free Dox with different Dox concentrations (0.01, 0.1, 0.5, 1, 5, 10, 20 and 40 μg Dox equiv./mL) in 10 μL of PBS were added and incubated in an atmosphere containing 5% CO_2 for 4 h at 37 °C. The medium was aspirated and replaced by fresh medium, and the cells were incubated for another 44 h. A PBS solution (5 mg/mL, 10 μL) of 3-(4,5-dimethylthiazol-2-yl)-2,5-diphenyl tetrazoliumbromide (MTT) was added and incubated for 4 h, the supernatant was carefully aspirated, and the MTT-formazan generated by live cells was dissolved in 150 μL of DMSO for 20 min. The absorbance at a wavelength of 570 nm was measured using a microplate reader. The cell viability (%) was determined by comparing the absorbance at 570 nm with control wells containing only cells. The experiments were performed in quartets.

The cytotoxicity of empty polymersomes with concentrations ranged from 0.5 to 2.0 mg/mL was determined similarly.

Flow cytometry assays and confocal microscopy measurements

The cellular uptake and intracellular drug release behaviors of cRGD-XIPs-Dox were studied in B16 cell using flow cytometry and confocal laser scanning microscopy (CLSM). For flow cytometry study, the cells were seeded in a 6-well plate (1×10^6 cells/well) using RPMI 1640 media containing 10% fetal bovine serum and antibiotics penicillin (100 IU/mL), and streptomycin (100 μg/mL) for 24 h. cRGD-XIPs-Dox, XIPs-Dox and free Dox in 500 μL PB was added (10.0 μg Dox equiv./mL). After incubation at 37 °C for 4 h, the cells were digested by 0.25% (w/v) trypsin and 0.03% (w/v) EDTA. The suspensions were centrifuged at 1000 × g for 3 min, washed twice with PBS, and then re-suspended in 500 μL PBS. Fluorescence histograms were then recorded with a BD FACS Calibur flow cytometer and analyzed using FCS express software. We analyzed 10,000 gated events to generate each histogram. The gate was arbitrarily set for the detection of red fluorescence. The cellular uptake of cRGD-XIPs-Dox in L929 fibroblasts were detected as above.

For CLSM study, B16 cells were cultured on microscope slides in a 24-well plate (1×10^5 cells/well) for 24 h, and cRGD-XIPs-Dox, XIPs-Dox and free Dox in 50 μL PB was added (10.0 μg Dox equiv./mL). The inhibitive experiments were performed by pre-treating B16 cells with 200 μg/mL free cRGD for 2 h prior to adding cRGD-XIPs-Dox. After 0.5, 1, 2 and 4 h incubation, the medium was removed and the cells on microscope plates were washed twice with PBS. The cells were then fixed with 4% paraformaldehyde solution for 15 min. The cytoskeleton and cell nuclei were stained with fluorescein isothiocyanate labeled phalloidin (phalloidin-FITC, green) and 4′,6-diamidino-2-phenylindole (DAPI, blue) for 1 h and 10 min, respectively. The fluorescence images were obtained using a confocal microscope (TCS SP5).

Dox calibration curves in the presence of blood or individual tissue

To establish standard curves of Dox in different tissues, 0.1 g of heart, liver, spleen, lung, kidney, and tumor tissue were weighed. For blood, 50 μL blood was used. 30, 15, 7.5, 1.5, 0.75, 0.15, and 0 μg Dox in 20 μL water were respectively added to different tissues and blood, and allowed to incubate overnight. To extract Dox, these samples were homogenized in 500 μL Triton X-100 (1%), and incubated with 1 mL DMF (containing 20 mM DTT) overnight at 4 oC. After centrifugation at 6000 rpm for 15 min, the supernatants were measured using a multi-function microplate reader (VARIOSKAN LUX, Thermo scientific, Ex 480 nm, Em 580 nm). The total fluorescence intensity of each sample subtracted the fluorescence of the Dox-free corresponding tissue gave the real fluorescence of Dox, and the latter was plotted against the Dox concentration yielding standard curves of Dox in the tissues individually.

Table S1. Characteristics of block copolymers

Entry	copolymer	M_n (kg/mol)			M_w/M_n[b] (GPC)	T_g[c] (°C)
		Design	^{1}H NMR[a]	GPC[b]		
1	PEG-P(DTC-IC)	5-(5-50)	5-(4.6-48.3)	63.2	1.31	-25.3
2	cRGD-PEG-P(DTC-IC)	6.5-(5-50)	6.5-(4.7-49.2)	68.3	1.23	-21.5

[a] Determined by ^{1}H NMR by comparing the integrals of signals at δ 3.04 (methylene proton of PDTC) with that of PEG methylene protons at δ 3.65 for MW of PDTC; and for MW of PIC, the integrals of signals e, f, b, c, and d in **Fig. S2** were compared and calculated according to the following equation: Ie/Ib = [(Ie+Ic)-Id]/{(Ib+If)- [(Ie+Ic)-Id]}.

[b] Determined by GPC (eluent: DMF, flow rate: 1 mL/min, standards: polystyrene, 30 °C).

[c] Determined by DSC.

Table S2. Calibration curves of free Dox in the presence of blood or each tissue

**Tissue**	**Standard Curves**	**Linear range (μg/mL)**	**R^2**
---	Y = 2.662 × X + 1.005	0.01-20	0.9911
Plasma	Y = 4.344 × X + 0.673	0.1-10	0.9963
Heart	Y = 3.808 × X - 0.194	0.5-20	0.9998
Liver	Y = 4.387 × X - 2.101	0.1-20	0.9975
Spleen	Y = 2.448 × X + 1.614	0.1-20	0.9956
Lung	Y = 3.008 × X - 1.748	0.1-20	0.9877
Kidney	Y = 4.668 × X + 0.360	0.1-20	0.9889
Tumor	Y = 2.892 × X - 0.591	0.1-20	0.9858

Table S3. Tumor-to-normal tissue (T/N) ratios of Dox at 4 h after *i.v.* injection

	Heart	Liver	Spleen	Lung	Kidney
cRGD-XIPs-Dox	7.27 ± 1.57	0.87 ± 0.19	1.44 ± 0.31	2.82 ± 0.61	1.94 ± 0.42
XIPs-Dox	2.88 ± 0.47	0.32 ± 0.05	0.57 ± 0.09	1.23 ± 0.20	0.74 ± 0.12
Free Dox	0.24 ± 0.06	0.08 ± 0.02	0.14 ± 0.03	0.29 ± 0.07	0.15 ± 0.04

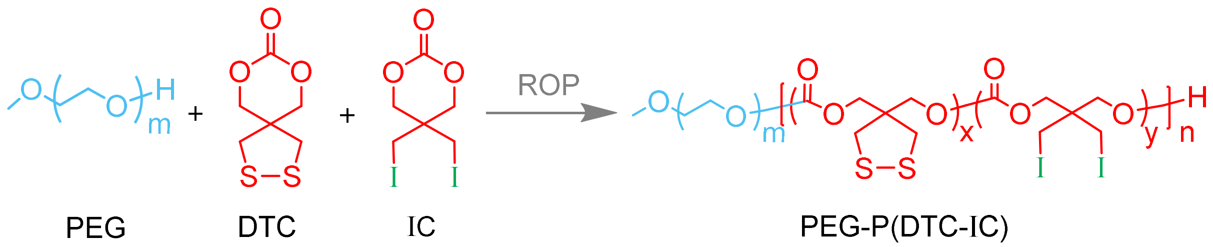

Figure S1. Synthesis of PEG-P(DTC-IC) by ring-opening polymerization. Conditions: zinc bis[bis(trimethylsilyl)amide], CH_2Cl_2, 40 °C, 3 days.

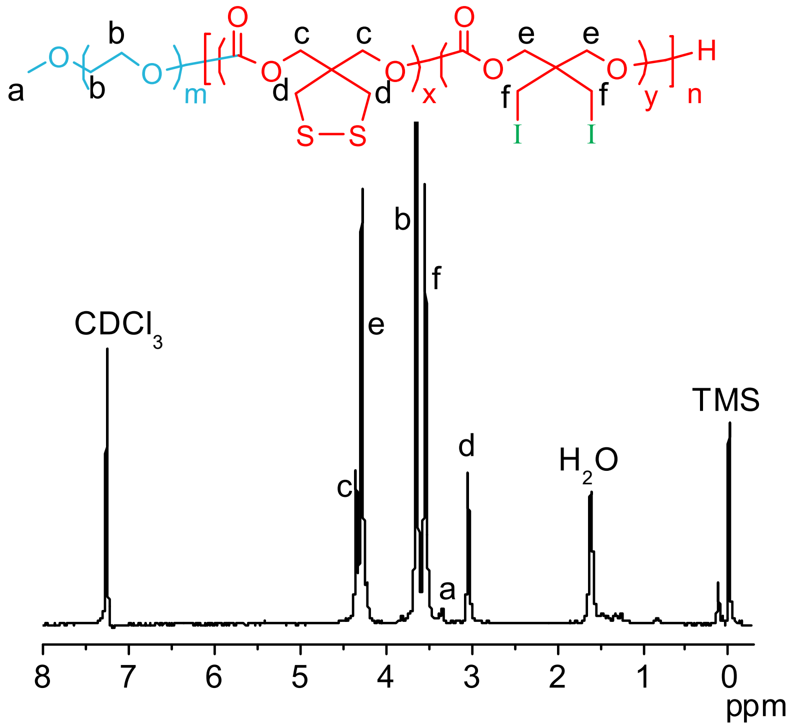

Figure S2. ^1H NMR spectrum (400 MHz, $CDCl_3$) of PEG-P(DTC-IC).

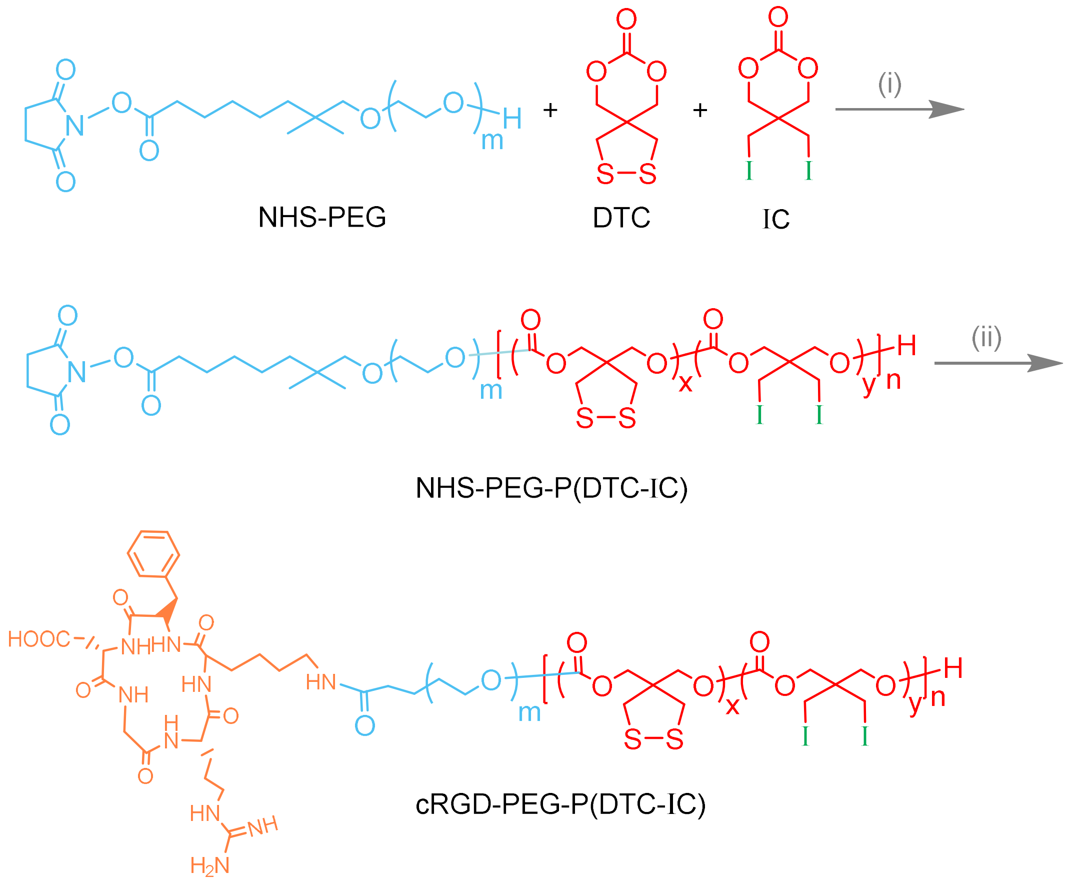

Figure S3. Synthesis of cRGD-PEG-P(DTC-IC). Conditions: (i) zinc bis[bis(trimethylsilyl)amide], CH_2Cl_2, 40 °C, 3 days; (ii) C(RGDfK), r.t, 48 h.

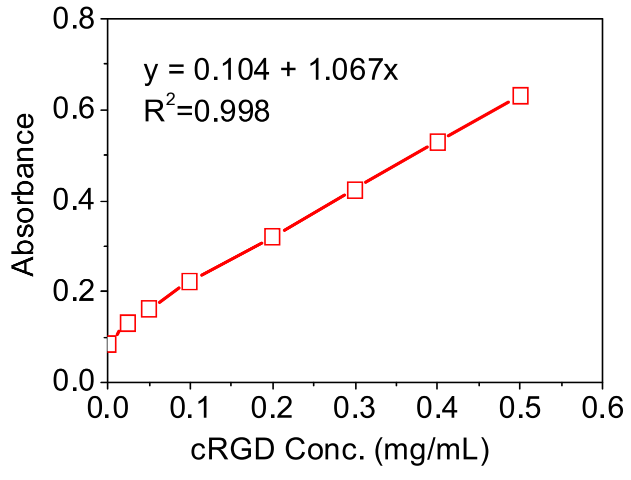

Figure S4. Standard curve of free cRGD peptide determined with the micro-BCA protein assay.

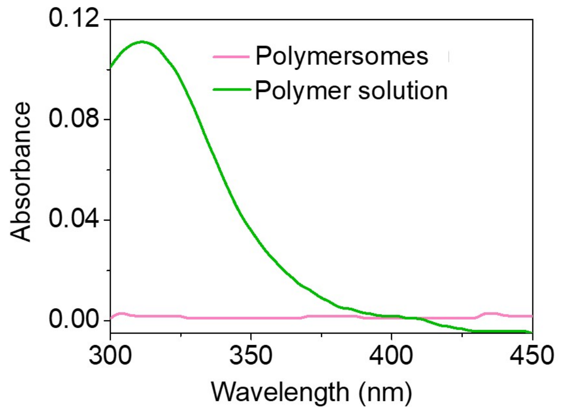

Figure S5. UV spectra of PEG-P(DTC-IC)/cRGD-PEG-P(DTC-IC) (8/2, mol/mol) polymer solution in DMSO and cRGD-XIPs dispersion at polymer concentration of 10 mg/mL.

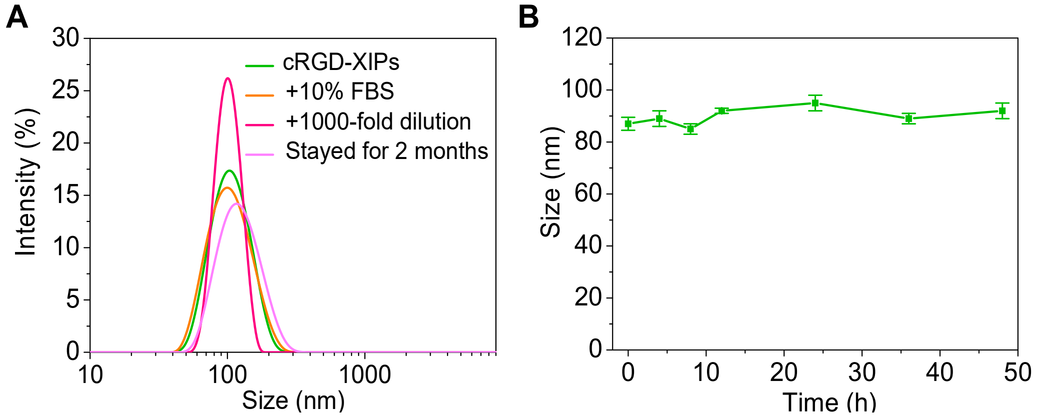

Figure S6. Size changes of cRGD-XIPs against 10% FBS, 1000-fold dilution and storage (**A**) and colloidal stability of cRGD-XIPs incubated in 10% FBS with time (**B**).

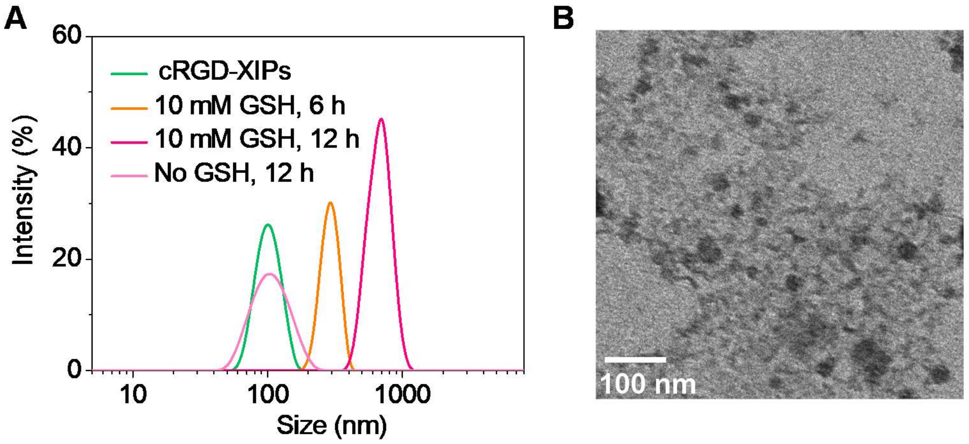

Figure S7. Size change of cRGD-XIPs in PB (pH 7.4, 10 mM) with or without 10 mM GSH at 37 °C (A) and TEM image of cRGD-XIPs treated with 10 mM GSH for 24 h (B).

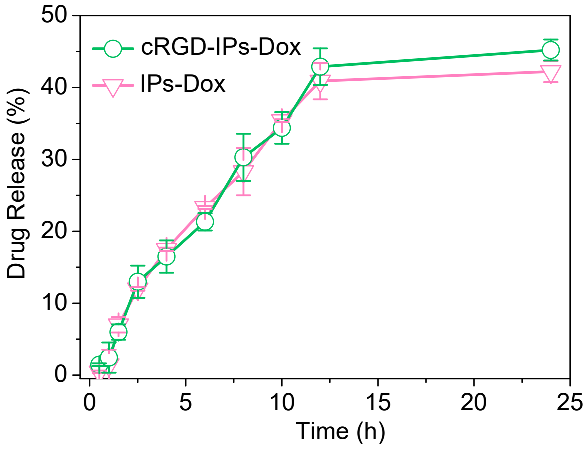

Figure S8. *In vitro* Dox release study of cRGD-IPs-Dox and IPs-Dox (non-crosslinked controls) in PB at 37 °C. Polymersome concentration was set 50 μg/mL.

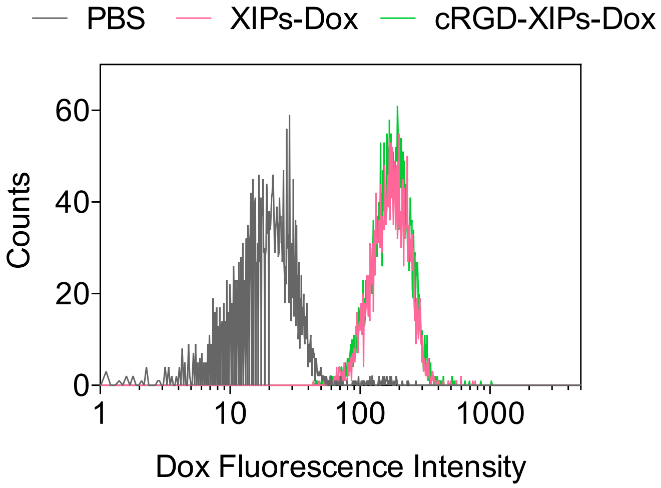

Figure S9. Flow cytometric analyses of L929 fibroblasts following 4 h incubation with cRGD-XIPs-Dox and XIPs-Dox (Dox: 10.0 μg/mL).

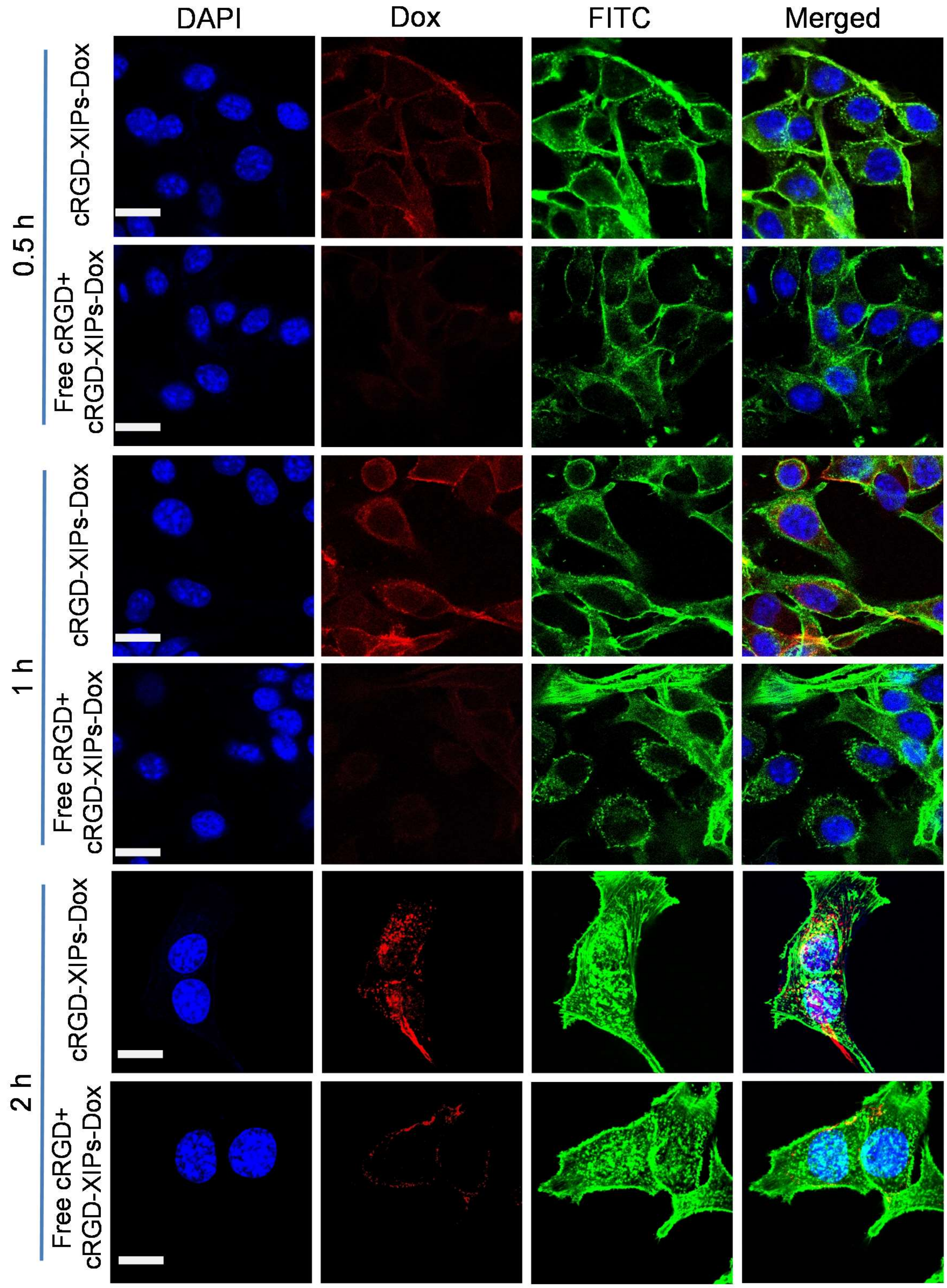

Figure S10. CLSM images of B16 cells following 0.5, 1 and 2 h incubation with cRGD-XIPs-Dox **(**Dox: 10.0 μg/mL). The inhibitive experiments were performed by pre-treating B16 cells with 200 μg/mL free cRGD for 2 h prior to adding cRGD-XIPs-Dox. The images from left to right are cell nuclei stained by DAPI (blue), Dox (red), cytoskeleton stained by phalloidin (green), and the merged images. Scale bars: 20 μm.

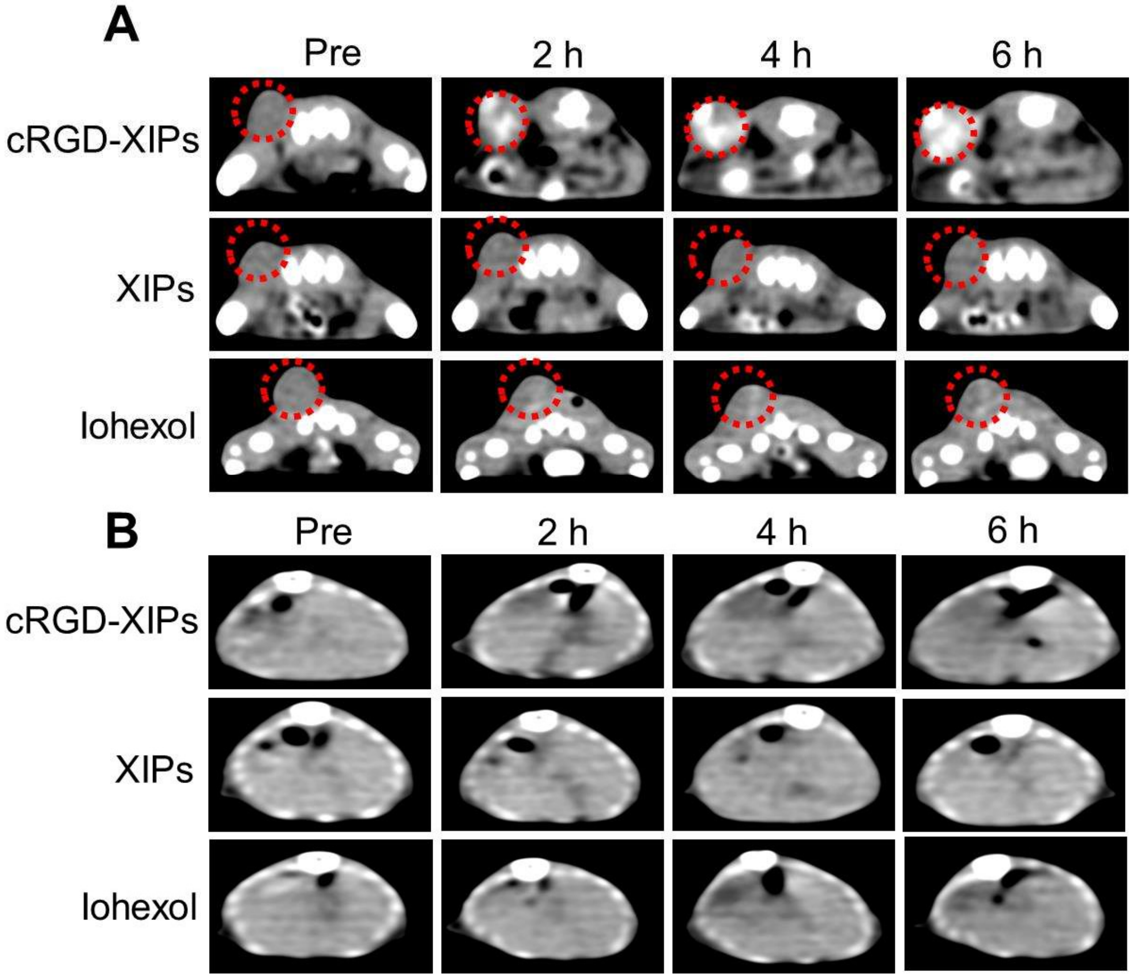

Figure S11. The axial CT images of the tumor (**A**) and liver (**B**) region of the mice before and at different time post *iv* injection of cRGD-XIPs, XIPs or iohexol. The red dotted circles indicate the tumor areas. The iodine dose was set at 350 mg I equiv./kg.

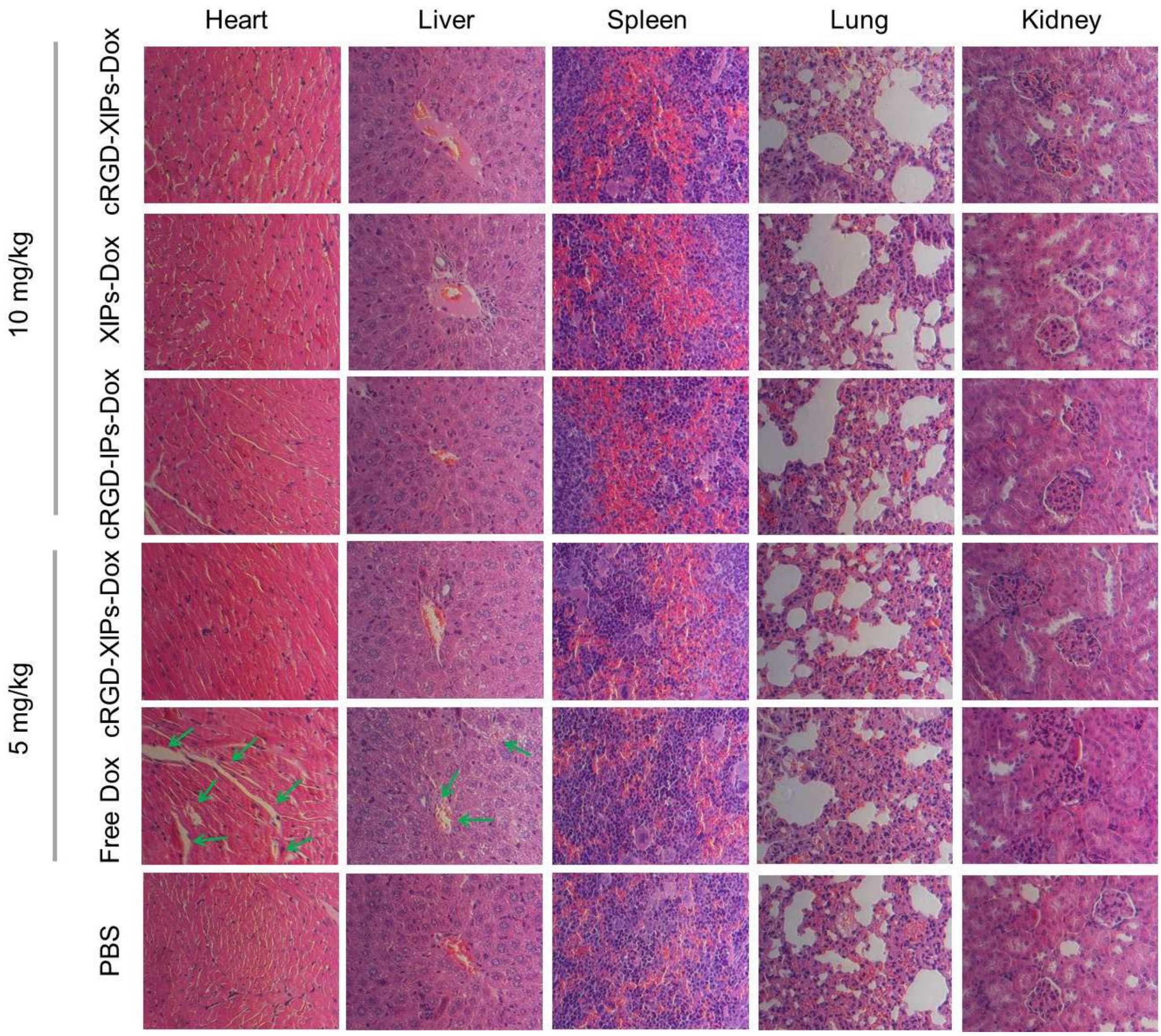

Figure S12. H&E stained organ slices of B16 tumor-bearing mice following 10 d treatment with cRGD-XIPs-Dox, XIPs-Dox, cRGD-IPs-Dox, free Dox or PBS. The images were obtained by a Leica microscope at 400× magnification.

References

1. Zou, Y.; Fang, Y.; Meng, H.; Meng, F.; Deng, C.; Zhang, J.; Zhong, Z., Self-crosslinkable and intracellularly decrosslinkable biodegradable micellar nanoparticles: A robust, simple and multifunctional nanoplatform for high-efficiency targeted cancer chemotherapy. J. Control. Release. 2016; 244: 326-35.
2. Zou, Y.; Wei, Y.; Wang, G.; Meng, F.; Gao, M.; Storm, G.; Zhong, Z., Nanopolymersomes with an ultrahigh iodine content for high-performance X-ray computed comography imaging in vivo. Adv. Mater. 2017; 29: 1603997.
